# Stress Signaling Responses in Plants

**DOI:** 10.1155/2016/4720280

**Published:** 2016-08-01

**Authors:** Marta Wilton Pereira Leite de Vasconcelos, Paloma Koprovski Menguer, Yanbo Hu, Luis Fernando Revers, Raul Antonio Sperotto

**Affiliations:** ^1^Universidade Católica Portuguesa, Centro de Biotecnologia e Química Fina (CBQF), Laboratório Associado, Escola Superior de Biotecnologia, Rua Arquiteto Lobão Vital, Apartado 2511, 4202-401 Porto, Portugal; ^2^Universidade Federal do Rio Grande do Sul, 91501-970 Porto Alegre, RS, Brazil; ^3^College of Life Science, Northeast Forestry University, No. 26 Hexing Road, Xiangfang District, Harbin 150040, China; ^4^EMBRAPA Uva e Vinho, Rua Livramento 515, 95701-008 Bento Gonçalves, RS, Brazil; ^5^Centro de Ciências Biológicas e da Saúde (CCBS), Programa de Pós-Graduação em Biotecnologia (PPGBiotec), Centro Universitário UNIVATES, Rua Avelino Tallini 171, 95900-000 Lajeado, RS, Brazil

Plants undergo continuous exposure to various biotic and abiotic stresses in their natural environment. To survive under such conditions, plants exhibit stress tolerance or stress avoidance through acclimation and adaptation mechanisms that ultimately reestablish cellular and organismal homeostasis or reduce episodic shock effects. These abilities involve intricate and complex mechanisms of perception, transduction, and transmission of stress stimuli, allowing optimal response to environmental conditions. The perception of stimuli and their expansion in cells involves signaling molecules such as intracellular calcium and reactive oxygen species, which intensify the action of particular signaling pathways. To date, the molecular mechanisms that are involved in each stress have been revealed comparatively independently, and so our understanding of convergence points between biotic and abiotic stress signaling pathways remains rudimentary. However, recent studies have revealed several molecules as promising candidates for common players that are involved in crosstalk between stress signaling pathways.

This special issue aimed to join original research and review articles related to understanding of plant responses to abiotic and biotic stress conditions, identifying novel players involved in plant responses to stress conditions, biotechnological strategies to increase plant tolerance to abiotic and biotic stresses, and understanding molecular interactions and crosstalks among different stress conditions.

M. Nourbakhsh-Rey and M. Libault in “Decipher the Molecular Response of Plant Single Cell Types to Environmental Stresses” explore* omic* studies to understand the response of single cell types to environmental stresses in order to clearly depict the contribution of each cell type composing the sample in response to stress. Cellular complexity of entire organs masks cell-specific responses to environmental stresses and logically leads to the dilution of the molecular changes occurring in each cell type composing the tissue/organ/plant in response to the stress. Specifically, the authors highlight that combining one or two* omic* analyses to look at single cell system biology can provide more precise molecular characterization and more dynamic models of the interactions between the plant and its environment.

J. Ren et al. in “Drought Tolerance Is Correlated with the Activity of Antioxidant Enzymes in* Cerasus humilis* Seedlings” describe the correlation between the activities of antioxidant enzymes and drought tolerance. By exploring drought-resistant and drought-susceptible* C. humilis* accessions, they compare the abilities of the contrasting genotypes to induce antioxidant defense under drought conditions. Their manuscript presents original data indicating that plants exhibiting a more efficient reactive oxygen species scavenging system in response to drought conditions have enhanced membrane protection, an ability that could be directly linked to their higher adaptation to drought.

X. Tang et al. in “Reference Gene Selection for qPCR Normalization of* Kosteletzkya virginica* under Salt Stress” employed RT-qPCR to select the most stable reference gene of the perennial halophytic plant* K. virginica* under salinity stresses. The stable reference gene selected in this study will be very helpful for revealing the gene expression profiles of* K. virginica* under salt stress, allowing a better understanding of the salt-resistant mechanisms in halophyte plants.

Y. Oono et al. in “Genome-Wide Transcriptome Analysis of Cadmium Stress in Rice” used RNAseq strategy to elucidate the molecular basis of the rice response to cadmium (Cd) stress, a widespread heavy metal pollutant that is highly toxic to living cells, negatively affecting nutrient uptake and homeostasis in plants. In this work, rice plants were hydroponically treated with low Cd concentrations, revealing novel Cd-responsive transporters by analyzing gene expression under different Cd concentrations. This study could help to develop novel strategies for improving tolerance to Cd exposure in rice and other cereal crops.

J. S. Rohila et al. in “Leaf Proteome Analysis Reveals Prospective Drought and Heat Stress Response Mechanisms in Soybean” investigate the effect of drought, heat, and cooccurring drought and heat stresses in the leaf proteome of two contrasting soybean genotypes. The authors identified genes involved in photosynthesis that were differentially expressed during drought and heat stress conditions, suggesting that photosynthesis-related proteins could be affecting RuBisCO regulation, electron transport, and Calvin cycle during abiotic stress, which ultimately alter the carbon fixation in leaves. The authors discuss the role of heat shock-related proteins and ROS detoxification capacity via carbonic anhydrase as heat and drought tolerance mechanisms, respectively.

J. M. Garcia-Mina et al. in “Involvement of Hormone- and ROS-Signaling Pathways in the Beneficial Action of Humic Substances on Plants Growing under Normal and Stressing Conditions” review our current knowledge about the mechanisms by which soil humus affects soil fertility. In particular, they discuss the relationships between two main signaling pathway families that are affected by humic substances within the plant: hormone- and ROS-mediated signaling pathways. The authors aim to integrate these events in a more comprehensive model and point out future research directions to unveil the complete mechanisms of regulation.

W. Fang et al. in “Cloning and Expression Analysis of One Gamma-Glutamylcysteine Synthetase Gene (Hb*γ*-ECS1) in Latex Production in* Hevea brasiliensis*” isolated a *γ*-ECS gene from the rubber tree and investigated its function linked to thiol content in latex. To understand the relation between *γ*-ECS and thiols and to correlate these findings to latex flow, the authors conducted RT-qPCR analysis and found that the expression levels of Hb*γ*-ECS1 were induced by tapping and ethrel stimulation, and the expression was related to thiols content in the latex. When looking at long-term flowing latex, the gene was related to the duration of latex flow. This work may have important biotechnological applications, since rubber tree is a major commercial source of latex, which is used for rubber production.

A. Vian et al. in “Plant Responses to High Frequency Electromagnetic Fields” conducted an original review, looking at the possible effects of HF-EMFs, which are increasingly present in the environment (due to, e.g., cell phones, Wi-Fi, and different types of connected devices) on different essential plant metabolic activities, such as reactive oxygen species metabolism, Krebs cycle, pentose phosphate pathway, chlorophyll content, and terpene emission. They found that not only are most of these pathways indeed modified by HF-EMF exposure, but also radiation brings about alterations in gene expression and plant growth. The authors propose to consider nonionizing HF-EMF radiation as a noninjurious, albeit influential environmental factor that induces significant changes in plant metabolism.

